# Proliferating toward sex: characterization of cell division of *Toxoplasma gondii*’s pre-sexual stages

**DOI:** 10.1128/mbio.02440-25

**Published:** 2026-05-19

**Authors:** Florencia Sena, Mohamed-Ali Hakimi, Maria E. Francia

**Affiliations:** 1 Laboratory of Apicomplexan Biology, Institut Pasteur Montevideo 123939, Montevideo, Uruguay; 2 Laboratorio de Bioquímica, Departamento de Biología Vegetal, Universidad de la República 585420 , Montevideo, Uruguay; 3 Institute for Advanced Biosciences (IAB), Team Host-pathogen interactions and immunity to infection, INSERM U1209, CNRS UMR5309, University Grenoble Alpes, Grenoble, France; 4 Unidad Académica de Parasitología y Micología, Facultad de Medicina, Universidad de la República 54795 , Montevideo, Uruguay; University of California Davis, Davis, California, USA

**Keywords:** apicomplexan, *Toxoplasma gondii*, cell division, merozoite, pre-sexual, endopolygeny, expansion microscopy

## Abstract

Toxoplasmosis is a disease of worldwide distribution, causing high morbidity and mortality in humans, as well as heavily impacting animal health and the economy. *Toxoplasma gondii*, the causative agent, is an intracellular parasite with a complex life cycle whose completion entails asexual, pre-sexual, and sexual stage conversions. Pre-sexual and sexual differentiation take place only within the intestinal epithelium of felines. Recently, several transcriptional factors and epigenetic components crucial to trigger parasite stage transitions within the cat have been identified, allowing, through precise genetic manipulation, obtaining pre-sexual stages known as merozoites *in vitro*. Through conditional depletion of two pre-sexual stage-specific gene silencing transcription factors, AP2XII-1 and AP2XII-2, we have characterized the interplay between cell division and the sequence of events leading up to differentiation of tachyzoites into merozoites. We explored genome duplication, assembly of daughter cells, karyokinesis, and cytokinesis, characterizing the differential cell division modes and kinetics undergone by critical structures along the differentiation axis. Building onto the pre-existing body of knowledge, primarily describing the underpinnings of these forms of division by transmission electron microscopy, our work contributes previously unexplored temporal and spatial resolution to the transitions between endodyogeny and endopolygeny, providing a conceptual framework for understanding and exploring *T. gondii*’s route of sexual differentiation.

**IMPORTANCE:**

Sexual development in *Toxoplasma gondii* is essential for transmission, but remains poorly understood, largely because pre-sexual stages are restricted to the feline intestine and have only recently become experimentally accessible. Here, we leverage an *in vitro* differentiation system to resolve how parasites transition toward merozoite formation at the cellular level. By combining expansion microscopy, stage-specific markers, and quantitative analyses, we define the temporal sequence of nuclear division and daughter cell assembly during merogony, addressing longstanding ambiguity regarding division modes in these stages. Our findings reveal that parasites can adopt alternative division strategies emerging from a polyploid intermediate, highlighting an unexpected degree of flexibility in how cell division is executed during differentiation. Beyond refining this developmental framework, this work establishes a foundation for future mechanistic studies of pre-sexual biology and provides broader insight into the diversity of eukaryotic cell division strategies.

## INTRODUCTION

Cell division is the central and universal process of life by which a mother cell divides into progeny bearing a complete complement of genetic and cellular material. Diverse modes of cellular proliferation have evolved to ensure maintenance or modulation of ploidy, as well as *de novo* generation or segregation of all functional cellular compartments, a basic requirement for every species’ survival. Apicomplexan parasites are obligate intracellular protozoa, causative agents of diseases that pose a tremendous burden to both animal and human health (1). Their exponential expansion within the cells of their hosts, mediated by high rates of proliferation, underlies their fundamental mechanism of pathogenesis (2). In addition, they represent exceptional models for studying divergent modes of division. They not only challenge the principles used by the best-studied mammalian cell division models but also provide a platform for studying the intersection of division and regulatory signals, as they showcase exceptional flexibility employing a diversity of cell division modes, adapting to developmental or environmental cues. Apicomplexans are known to divide by endodyogeny, endopolygeny, schizogony, and/or binary fission (3). However, our fundamental understanding of the molecular underpinnings and variability among Apicomplexans’ modes of cell division remains succinct, with only a few species and a handful of life stages having been thoroughly explored. In particular, how different cell division modes co-exist and crosstalk in each species remains fairly unexplored.

*Toxoplasma gondii*, the causative agent of toxoplasmosis, is an opportunistic pathogen of immunocompromised individuals and a major cause of acquired congenital disease (4, 5). One third of the world population is chronically infected, and two hundred thousand new cases of congenital toxoplasmosis are accumulated yearly worldwide, entailing a cumulative burden of 1.2 million daily adjusted life years (DALYs) (4).

Asexually replicating *T. gondii* life forms, the tachyzoite and bradyzoite, are the major clinically relevant life forms of the parasite (6). Tachyzoites are fast proliferating, while bradyzoites remain metabolically active but proliferate at a significantly lower rate (7, 8). Tachyzoites and bradyzoites divide by endodyogeny. This mode of division entails the duplication of genetic material by semi-closed mitosis, and the internal assembly of two fully invasion-competent daughter cells in the cytosol of the mother (3, 9, 10). Among other peculiarities, *T. gondii* asexual division is locally regulated by the centrosome, which, in addition to fulfilling its canonical role in mitotic spindle assembly during division, organizes chromosomes throughout the cell cycle and acts as a platform for the assembly of new daughter cell scaffolds (11).

Upon infection of the intestinal epithelium of the definitive host (felids), *T. gondii* undergoes a marked change in its biology, transitioning through a series of pre-sexual developmental stages culminating in the production of merozoites within the small intestine (12). Terminal merozoites undergo a limited number of replication cycles (two to four rounds) before differentiating into micro- and macro-gametes (13). Fusion of these gametes leads to oocyst formation, a process essential for genetic recombination and the production of environmentally resistant stages that facilitate widespread parasite transmission (14).

Merozoite formation occurs within 2 days of colonizing the feline intestine. Reaching the mature merozoite stage entails parasites to asynchronously move through five developmental states (12). These morphologically distinct entero-epithelial phases, named EES 1-5, correspond to very early, early, mixed, late, and very late stages following the cat’s oral infection. These forms have also been independently described as morphotypes A through E (15). Although EES 1-5 could broadly correlate with morphotypes A–E, the two classifications were established based on different sampling times and criteria and therefore are not strictly equivalent.

Our understanding of the morphological transitions experienced by pre-sexual and sexual life forms has been advanced by observations using transmission electron microscopy of infected cat’s tissues fixed at given time points after experimental oral infections. Pioneering work by Dubey and Frenkel in the 70s described that EES 1-5 replicate by endopolygeny (16). Further work elucidated that this mode of division entails successive rounds of DNA duplication, with geometric nuclear expansion, and a final round of internal daughter cell assembly, generating 8–16 progeny per replication cycle (17). Though these structural insights have been critical to grasping the morphological complexity of these experimentally inaccessible life stages, our mechanistic understanding of their cell division and stage-transition mechanisms has been thus far limited by the lack of detailed cellular level insight. How nuclear material is duplicated, parceled, and segregated, although coordinated with the assembly of daughters along time, and how this is intertwined with stage transitions, remain ill-explored. In particular, the sequence of events leading up to mature merozoites remains unclear.

Transition through stages is regulated at the transcriptional and epigenetic level. Apetala-related transcription factors, ApiAP2 transcription factors, have been shown to function as key negative regulators of pre-sexual commitment and merogony, primarily through their association with the HDAC3/MORC epigenetic repressor complex (18). Conditional depletion or knockdown of these transcription factors leads to the derepression of pre-sexual- and sexual-associated gene programs, inducing merozoite formation *in vitro* (19–24). Specifically, AP2XI-2 and AP2XII-1 are constitutively expressed in asexual stages and suppress the transcription of merozoite-, bradyzoite-, and sexual stage-specific genes by binding promoter regions enriched at transcription start sites and promoting chromatin compaction (21). In addition to providing a mechanistic framework to transcriptional and epigenetic regulation of pre-sexual commitment, recent studies have granted unprecedented *in vitro* access to pre-sexual forms of *T. gondii*, bypassing the imperative need to use felids as experimental models. In turn, this tool provides a framework to quantitatively dissect the spatial and temporal dynamics of the division machinery during merozoite development. These processes have not been characterized in sufficient detail and lack temporal resolution, precluding a direct link between proliferation and differentiation along the tachyzoite–merozoite axis.

Herein, we capitalize on inducible life-stage transitioning strains to assay the dynamic changes undergone by nuclear, cytoplasmic, and membrane components critical for cell division progression, along with differentiation. We establish the chronology of successive events leading up to terminal pre-sexual differentiation, finely mapping how critical structures such as the centrosome, the kinetochores, and the daughter cell scaffolds interplay in time and space as merozoites progress toward their final destination, sex.

## MATERIALS AND METHODS

### Parasite cell culture

*T. gondii* RH AP2XII-1–mAID–HA/AP2XI-2–mAID–Myc strain (21) was maintained in Vero cells (kidney of an African green monkey, ATCC CCL-81). hRPE (human retinal pigmented epithelial, ATCC CRL-4000) cells were used as host cells for all immunofluorescence assays. Parasites and host cells under sterile conditions using a biosafety level 2 cabinet were grown in Dulbecco modified Eagle medium (Gibco) supplemented with 10% fetal bovine serum (Gibco), 4 mM L-glutamine (Gibco), 200 U/mL of penicillin, 200 mg/mL of streptomycin (Gibco), and 0.5 mM indoleacetic acid (IAA; I5148, Sigma-Aldrich) when indicated. Cultures were incubated in a humidified chamber at 37°C and 5% CO_2_.

### Immunofluorescence assay (IFA)

hRPE cells were grown to 90% confluency on 18 mm glass coverslips and then infected with 1 × 10^8^ extracellular parasites. Intracellular parasites were fixed at the indicated time points using cold (−20°C) methanol for 5 min, washed with 1× phosphate buffered saline (PBS), and blocked in 1× PBS and 3% bovine serum albumin (BSA) for 10 min at room temperature. Primary and secondary antibodies were prepared on 1× PBS and 3% BSA and incubated at room temperature and in the dark for an hour each. Primary antibodies used include mouse anti-GRA11b at 1:200 (25; kindly provided by Dr. Chandra Ramakrishnan, University of Zurich), rabbit anti-ROP26 at 1:1,000 (21), rabbit anti-GRA80 at 1:1,000 (21), mouse anti-centrin at 1:500 (Cell Signaling, 04-1624), mouse monoclonal anti-IMC-1 45.5 at 1:500 (26; kindly provided by Dr. Gary Ward, University of Vermont), guinea pig anti-TgNDC80 at 1:2,000 (27; kindly provided by Dr. Marc-Jan Gubbels, Boston College), rabbit anti-TgH2Bz at 1:500 (28; kindly provided by Dr. Laura Vanagas, INTECH-Chascomus), rabbit anti-Mys at 1:2,000 (29; kindly provided by Dr. Lilach Sheiner, University of Glasgow), and mouse anti-acetylated tubulin at 1:1,000 (Sigma, catalog no. T7451). The fluorescent dye Alexa Fluor 488 NHS Ester (Succinimidyl Ester) at 1:20,000 (Thermo Fisher, A20100). Secondary antibodies include goat anti-mouse Alexa Fluor 488 (Invitrogen, A28175), goat anti-rabbit Alexa Fluor 488 (Invitrogen, A-11008), goat anti-guinea pig Alexa Fluor 594 (Invitrogen, A-11076), goat anti-rabbit Alexa Fluor 647 (Invitrogen, A27040), and goat anti-mouse Alexa Fluor 647 (Invitrogen, A-21235). All secondary antibodies were used at a dilution of 1:2,000. Finally, coverslips were washed three times with 1× PBS for 10 min and mounted onto ProLong Glass Antifade mounting with NucBlue (Invitrogen, P36985).

### DNA content and parasite proliferation determination by flow cytometry

hRPE cells were infected with approximately 10^7^ parasites pre-stained with 10 µM Carboxyfluorescein diacetate succinimidyl ester dye (CFSE, Abcam ab113853) for 10 min at 37°C protected from light. After 24–48 h of infection with or without 0.5 mM IAA addition, infected host cells were collected from the surface of culture T-25 flasks using a cell scraper. Cells were mechanically disrupted by passing the suspension through a 27-gauge syringe needle at least 10 times to release intracellular parasites. The lysate was then passed through a 3 μm pore size polycarbonate filter to remove host cell debris. The filtrate was centrifuged at 400 × *g* for 10 min at 25°C to pellet extracellular parasites and washed with 1× PBS, followed by a second centrifugation at 400 × *g* for 10 min at 25°C to recover extracellular parasites. Filter-purified parasites were fixed in cold methanol for 5 min, pelleted at 400 × *g*, suspended in 1× PBS, and stained with 200 µg/mL Propidium Iodide (PI) and 0.2 mg/mL RNase cocktail (Zymoresearch) in the dark at room temperature for 30 min. Flow cytometry data acquisition and analysis were performed using an Attune NxT flow cytometer (Thermo Fisher Scientific) and FlowJo v.10.10.0 software, respectively. CFSE and PI fluorescence was detected by exciting lasers at 488 nm (BL1) and 561 nm (YL2), collecting emission with band-pass filters of 530/30 and 620/15 nm, respectively. For each sample, a minimum of 10,000 events gated on a FSC versus SSC dot plot and excluding doublets were recorded.

### Ultrastructure expansion microscopy (U-ExM)

Ultrastructure expansion microscopy was performed as described previously for *T. gondii* tachyzoites (30) with the following modifications: 13 mm glass coverslips coated with hPRE cells and infected with RH AP2XII-1–mAID–HA/AP2XI-2–mAID–MYC parasites (without or with addition of IAA) were fixed with cold methanol as described for IFA. Infected and fixed coverslips were incubated overnight with 2% acrylamide and 1.4% formaldehyde at 37℃. Gelation was induced by adding the monomer solution (19% sodium acrylate, 10% acrylamide, 0.1% BIS-acrylamide, and freshly added 0.5% tetramethylethylenediamine and 0.5% ammonium persulfate in 10× PBS) onto the coverslip at 37℃ for 1 h. Denaturation was induced by incubation with a denaturalization buffer (200 mM SDS, 200 mM NaCl, 50 mM Tris pH 9) for 1.5 h at 95℃. The first round of expansion was done in ultrapure water for 30 min. A small piece of gel was cut to proceed with the labeling. Gels were blocked on 1× PBS and 2% BSA for 30 min at room temperature, and the primary antibody was incubated overnight at room temperature on 2% BSA in PBS 1×, or the fluorescent dye NHS ester 488 nm was incubated on PBS 1× for 1 h. Following three washes in PBS and 0.1% Tween with agitation, gels were transferred to secondary antibodies incubated in 2% BSA in PBS 1× for 2 h at room temperature, protected from light. Following three washes in PBS and 0.1% Tween with agitation, gels were subjected to a final round of expansion by incubating antibody-labeled gels in ultrapure water 2 times for 30 min each. Gels were mounted onto a MatTek plate previously incubated with 0.1 mg/mL of Poly-D-Lysine (Gibco, A3890401) to avoid drifting. Imaging was carried out within a maximum of 3 days post-staining.

### Confocal microscopy and imaging processing

All images were acquired using a Zeiss confocal LSM800 microscope using a Plan-Apochromat immersion oil 63× lens with a numerical aperture of 1.40. Alexa fluor antibodies and fluorescent dyes were excited using the 405 nm diode, 488 nm Argon, 561 nm solid-state, and 633 nm HeNe lasers. All images were acquired and processed using Zeiss ZEN blue edition v3.9 software. All measurements and counts were carried out using Fiji (31). Images were deconvolved using Huygens Professional v19.10.0p2 64b (Scientific Volume Imaging, the Netherlands).

## RESULTS

The conditional and simultaneous depletion of AP2XI-2 and AP2XII-1, upon the addition of indole-acetic acid (IAA), to the RH AP2XII-1–mAID–HA/AP2XI-2–mAID–Myc (hereafter referred to as AP2XII-1/AP2XI-2_iKD) strain leads to a change in the strain’s transcriptional program promoting a full transition from tachyzoites to merozoites *in vitro,* recapitulating pre-sexual *T. gondii* development (21). Antunes and colleagues described that tachyzoite to merozoite transitions entail marked changes in the secretory profiles of these parasites whereby ROP26 was reported to mark bradyzoite and pre-merozoite stages, while GRA11b and GRA80 are expressed hierarchically. GRA11b/GRA80 simultaneous expression is restricted to mature *in vitro* merozoites. Combinations of these stage-specific markers can be used to define the presence of specific developmental stages *in vitro*. Early differentiation stages*,* likely corresponding to Morphotypes B, are defined by ROP26+/GRA11b− expression, while more differentiated morphotypes C and D showcase ROP26+/GRA11b+ expression. Finally, mature merozoites are defined by GRA11b+/GRA80+ (21). To characterize the relative abundances of each stage under our experimental conditions, we tracked *in vitro* merogony progression over time (Fig. S1A). In line with previous findings, we observed that untreated parasites express neither ROP26 nor GRA80 or GRA11b. Twelve hours after IAA addition, 20% of the tachyzoite population has transitioned into morphotype B (ROP26+/GRA11b−); 50% of the population can be found as morphotype C and D, 24 and 36 h post-induction, respectively. By 48 h, 80% of the parasites express the merozoite GRA11b+/GRA80+ signature. Despite the progressive enrichment of specific forms, distinct stages of differentiation co-exist at any given time point (Fig. S1B).

**Fig 1:**
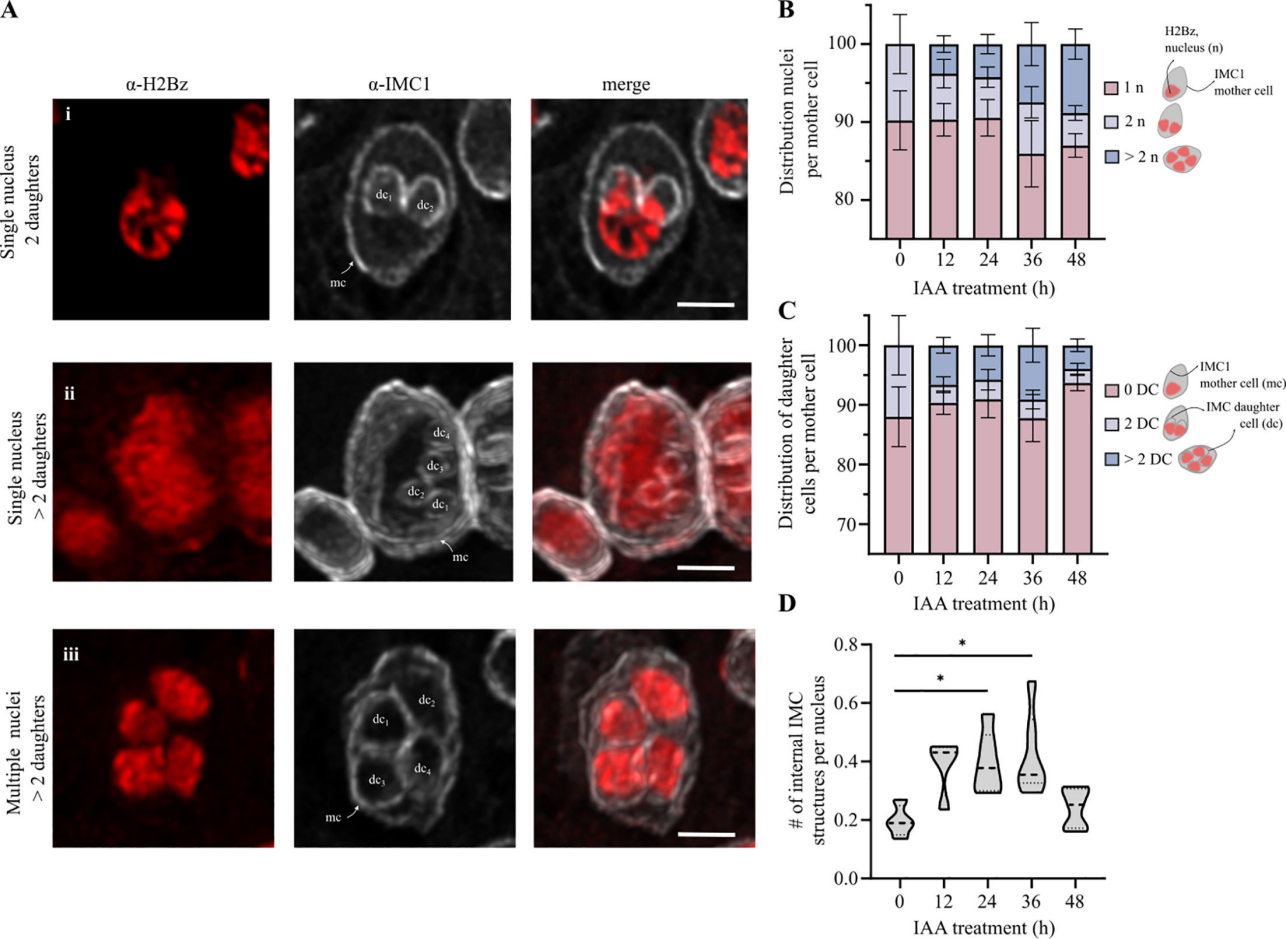
Characterization of nuclear and daughter cell assembly upon depletion of AP2XII-1 and AP2XI-2. (**A**) Representative images of parasites stained with anti-IMC1 (parasite membrane marker, gray) and anti-H2Bz (nuclear marker, red) reveal heterogeneous combinations of nuclear number and associated daughter cell formation patterns following AP2XII-1 and AP2XI-2 depletion. dc, daughter cells; mc, mother cell. All images correspond to selected z-slices from the acquired z-stacks. The scale bar represents 2 μm. (**B**) Distribution of cells exhibiting a given number of nuclei (n) packed into an IMC1 + mother pre (−IAA) and post IAA (+IAA) addition to the growth media. (**C**) Distribution of a given number of daughter cells (dc) packed into a mother (mc) pre (−IAA) and post (+IAA) addition at the indicated time points. The number of daughter cells was quantified as the total number of internal IMC structures within a single mother cell. (**D**) The truncated violin plots represent the total number of daughter cells packed per nucleus of a given mother cell over time upon IAA addition to the growth media. A violin plot represents the data distribution, with the median indicated by the central dotted line. Note that given the heterogeneous mixture of dividing and non-dividing parasites in the culture, the number of internal IMC1 structures per nucleus ranges between 0.2 and 0.6. Quantification was performed on 10 randomly selected fields per three biological replicates. Asterisks indicate statistically significant differences (ANOVA test), *P < 0.05.

Using these time points as a reference, we set out to explore the temporal interplay between daughter cell assembly and nuclear division, along with differentiation by indirect immunofluorescence assay (IFA). Inner membrane complex (IMC) proteins are hierarchically laid out, giving rise to new daughters within the mother (26). Using an anti-IMC1 antibody in combination with a nuclear marker (the histone H2Bz), we were able to observe a plethora of nuclear configurations following AP2XII-1/AP2XI-2_iKD depletion and its synchrony or lack thereof with daughter cell assembly (Fig. 1A). Parasites displaying duplicated nuclei associated with the assembly of two daughter cells, consistent with a canonical endodyogeny-type of division, could be observed (Fig. 1A, i). We also observed parasites displaying a seemingly enlarged single nucleus, which concomitantly coexisted with multiple budding daughter cells (Fig. 1A, ii). Finally, multinucleated parasites emerge. These bear multiple discrete nuclei spatially organized within a common cytoplasm and assemble multiple internal daughters (Fig. 1A, iii). These distinct architectural states suggest dynamic transitions entailing distinct dynamics of coupling and uncoupling of karyokinesis and cytokinesis, with parasites exhibiting features consistent with an endopolygeny-like replication. We next set out to determine whether these observable states corresponded to specific morphotypes. For this, we quantified both the number of nuclei per mother cell as well as the total number of mothers assembling daughter cells at a given time post-AP2XII-1/AP2XI-2_iKD depletion. While only either a single or a duplicated nucleus is observable prior to IAA addition, multiple nuclei per mother are detectable upon IAA addition, with a particular enrichment of multinucleated cells by 36 and 48 h post-IAA addition (Fig. 1B). As expected, prior to AP2XII-1/AP2XI-2_iKD depletion, daughter cell formation was consistently limited to two per mother cell. In turn, more than two daughter cells inside a mother cell were distinguishable at later time points after co-depletion. We observed that up until 36 h post-depletion, the number of cells bearing more than two daughter cells increases significantly. By 48 h, this number reduces significantly (Fig. 1C). Additionally, we quantified the average number of assembling daughter cells per nucleus. In endodyogeny, this ratio varies between 0 and 1, with 0 corresponding to no daughter cell assembling and one nucleus, and one corresponding to the ratio between two daughter cells and two segregated nuclei. Given that only approximately 15% of tachyzoites actively assemble daughters, the median falls well below 1 (approx. 0.2; Fig. 1D). Upon IAA addition, the ratio between daughter cells and nuclei increases significantly, reaching its maximum by 24 and 36 h post-AP2XII-1/AP2XI-2_iKD depletion (Fig. 1D). However, taken together, these data suggest different scenarios for these two time points. On one hand, by 24 h post-AP2XII-1/AP2XI-2_iKD, the number of multinucleated cells does not increase significantly; however, mothers bearing more than two daughter cells are readily detectable. The increased ratio in this scenario could be given by an increase in the number of daughter cells assembled, without the concomitant nuclear mitosis. Conversely, by 36 h, more than two daughter cells and multiple nuclei are simultaneously detectable, thereby increasing the number of events whose ratio approaches 1. To test these scenarios, we next assessed parasite ploidy and the dynamics of cytokinesis.

**Fig 2:**
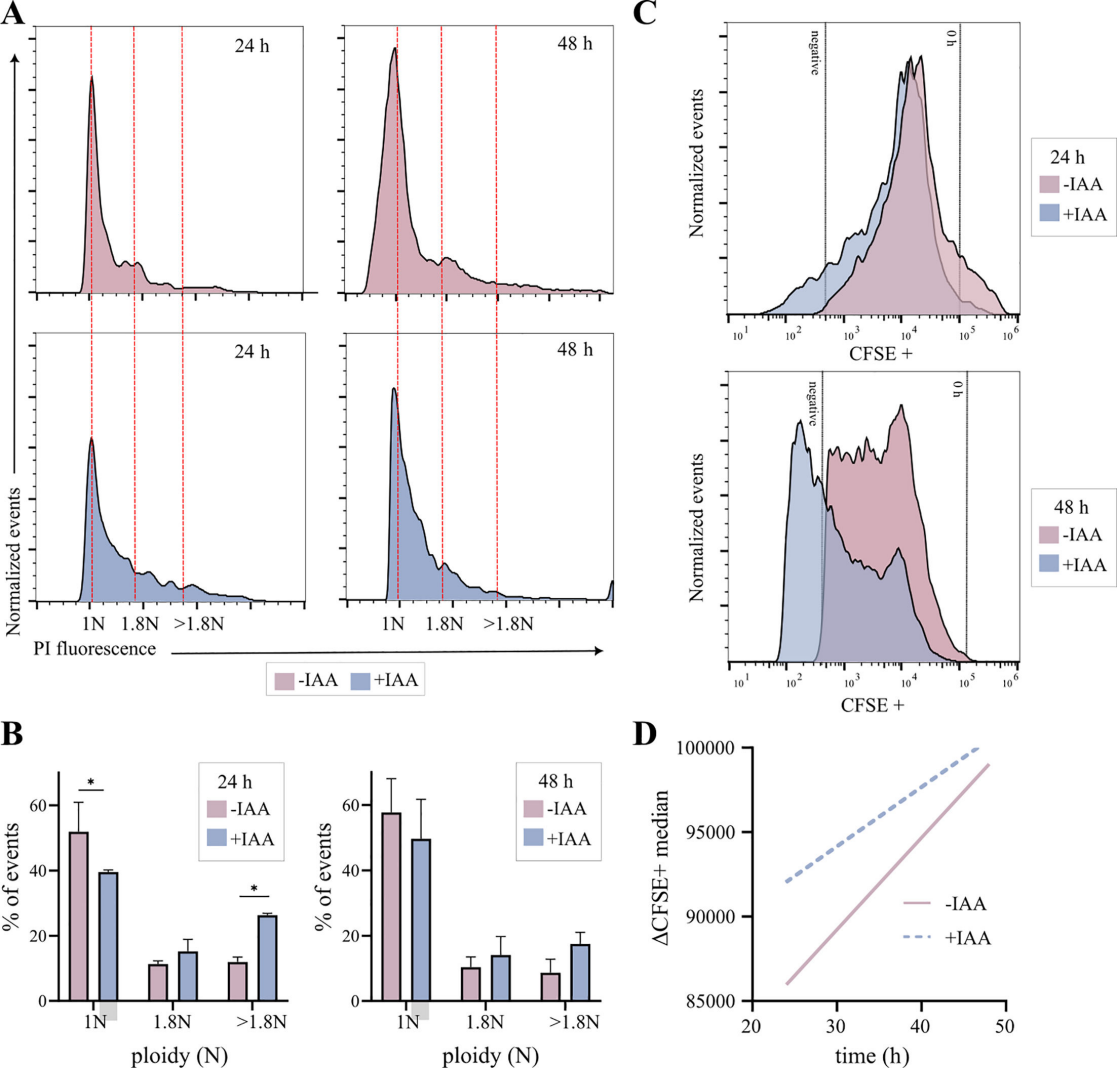
Ploidy and parasite proliferation determination by flow cytometry upon depletion of AP2XII-1 and AP2XI-2. (**A**) Flow cytometric analysis of parasites stained with propidium iodide after RNase treatment with or without IAA treatment for the indicated times. Doublets were excluded by singlet gating prior to analysis. For each sample, 10,000 parasites were analyzed (*y* axis) using a YL-2 linear scale; fluorescence values corresponding to 1N, 1.8N, and more than 1.8N DNA content are indicated on the *x* axis and marked by vertical dashed red lines. (**B**) DNA content histograms were used to define 1N, 1.8N, and > 1.8N populations based on PI fluorescence intensity. Bar graphs represent the percentage of total singlet events within each ploidy category under the indicated conditions. Data shown corresponds to the mean ± SD from two independent experiments. Asterisks indicate statistically significant differences (Student’s test), *P < 0.05. (**C**) Flow cytometric analysis of CFSE fluorescence measurements represented as histograms. Cells were pre-loaded with CFSE and cultured in the absence or presence of IAA for the indicated durations. Doublets were excluded by singlet gating prior to analysis. Each sample analyzed 10,000 parasites using a BL-1 log scale. CFSE fluorescence progressively decreased over time, consistent with dilution of the dye through successive cell divisions. Unlabeled cells included in the analysis as a negative control correspond to the fluorescence intensity marked by the dashed line named negative. The black-dotted line labeled 0 h represents the initial CFSE fluorescence intensity of parasites immediately after dye loading. Histograms were normalized using the mode to allow comparison of the distribution across time points. (**D**) Median CFSE fluorescence intensity of CFSE-positive cells at the indicated time points under −IAA and +IAA conditions. Median values were calculated from each gated CFSE-positive population.

To directly assess parasite ploidy, we quantified DNA content using propidium iodide (PI) staining followed by flow cytometry. Prior to IAA addition, parasites displayed the expected predominance of a 1N population corresponding to cells in G1, with a minor fraction of cells displaying the 1.8N expected ploidy for dividing cells (32). Virtually no events were detectable beyond 1.8N (Fig. 2A). In contrast, co-depletion of AP2XII-1 and AP2XI-2 resulted in a clear redistribution of DNA content; 24 h post IAA, a reduction in the 1 N population was accompanied by an increased proportion of parasites with 1.8N DNA content and the emergence of a >1.8N population. Quantification of the percentage of total events within each category confirmed a significant decrease in 1N parasites and a concomitant increase in both 1.8N and >1.8N populations upon co-depletion (Fig. 2B). Notably, the expansion of the >1.8N fraction over time supports the notion that loss of AP2XII-1 and AP2XI-2 promotes DNA over-duplication and an increase in ploidy. Consistent with prior observations, DNA content did not significantly differ from that of tachyzoites by 48 h post-depletion, when merozoites have reached maturity. To simultaneously analyze parasite proliferation, we independently monitored Carboxyfluorescein succinimidyl ester dye (CFSE) dilution over time in pre-loaded parasites. CFSE dilution over time serves as a proxy of cytoplasmic division, as it decreases in a stepwise manner across successive rounds of cell division (33). In particular, cells exhibiting altered DNA content under IAA treatment displayed markedly distinct kinetics of CFSE dilution, with a large proportion of the population retaining higher levels of fluorescence by 24 h when compared to their endodyogeny-dividing counterparts. Changes in ploidy were therefore accompanied by a reduction in cytokinesis (Fig. 2C). In contrast, by 48 h, the levels of CFSE fall below the detection threshold more pronouncedly for +IAA-treated parasites than for untreated ones, likely reflecting a greater number of cellularization events leading up to individuals at this time point (Fig. 2D). Together, PI-based flow cytometry and CFSE dilution analyses revealed a progressive increase in nuclear ploidy that was accompanied by reduced parasite cytoplasm division by 24 h. This trend reverses at 48 h, with most parasites returning to a 1N ploidy and arising from an increased number of cytokinesis rounds.

**Fig 3:**
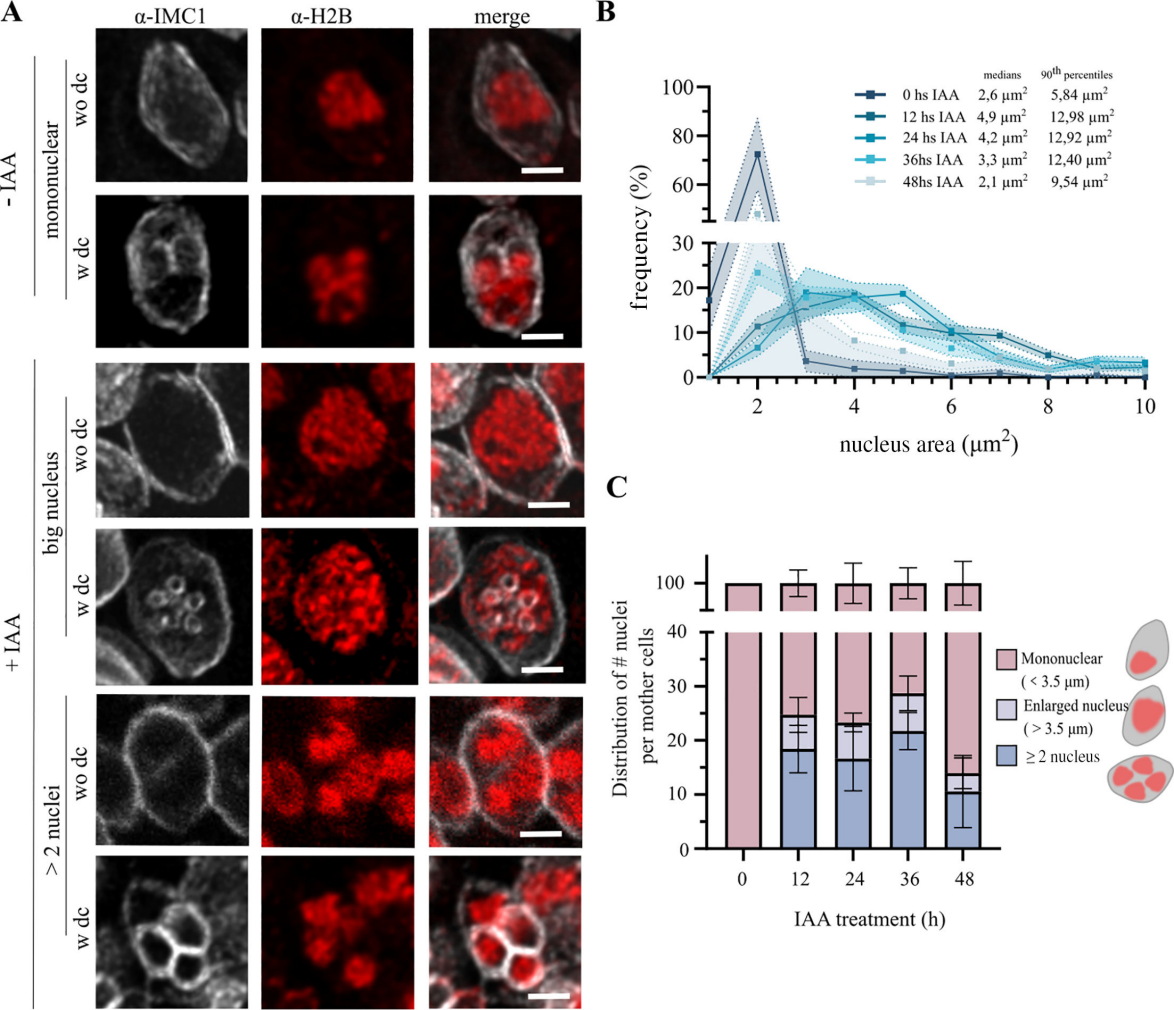
Nuclear dynamics following co-depletion of AP2XII-1 and AP2XI-2. (**A**) Representative IFA of each nuclear dynamic detected showing IMC1 (membrane marker; gray) and H2Bz (nuclear marker; red) without (−IAA) or with (+IAA) depletion of AP2XII-1 and AP2XI-2. dc represents with (w) or without (wo) daughter cell presence. Scale bars all represent 2 µm. (**B**) The plots represent the distribution of frequencies of the nuclei area of parasites at each time point. Within the color references, the median value and the 90th percentiles of each plot are represented. The colored shaded areas show the standard error of the mean. Approximately 40 nuclei were counted from at least three independent experiments. (**C**) Distribution of the number of nuclei (H2Bz) packed into a mother cell (IMC) was counted as mononuclear (less than 3.5 µm), presence of an enlarged nucleus (more than 3.5 µm), or multiple nuclei (defined by the presence of more than two nuclei) independently of the assembly of daughter cells. The averages are plotted with the respective standard error of the mean. Quantification was performed on 10 randomly selected fields per three biological replicates.

To further characterize the mechanisms underlying these transitions, we categorized distinct parasite morphologies, quantifying changes in nuclear size and organization. We frequently observed a heterogeneous population of co-existing stages in which parasites displayed duplicated and segregated nuclei, with or without concomitant daughter cell formation (Fig. 3A; +IAA: “more than two nuclei”). In addition, we consistently observed mother cells showcasing a single nucleus perceivably larger (Fig. 3A; +IAA: “enlarged nucleus”). To quantitate these observations, we measured relative nuclear area over time, which highlighted an increase in the median nuclear area and a marked elevation of the 90th percentile (expansion of the upper distribution tail), indicating accumulation of enlarged nuclei over time. While at 0 h, the majority of nuclei exhibited a size of around 2.5 μm^2^, consistent with that reported size for a tachyzoite (34), by 12 and 24 h of IAA addition, nuclei showed a significant increase, doubling to approximately 5 μm^2^. By 36 and 48 h, nuclear size progressively decreased (Fig. 3B). Statistical analysis (Kolmogorov–Smirnov test) revealed a significant shift in the area of individual nuclei between 0 and 12 h (D = 0.2843, *P* < 0.05), as well as at 36 h (D = 0.3805, *P* < 0.01). To quantitatively assess the occurrence of changes in nuclear organization, we categorized parasites based on the number and morphology of H2Bz-positive nuclei contained within a single IMC-defined mother cell. Parasites were scored as mononuclear (i.e., containing a single nucleus of <3.5 µm^2^) or containing a single enlarged nucleus (>3.5 µm), or multinucleated (defined as more than one discrete nucleus), independently of daughter cell assembly status. Under −IAA conditions, the vast majority of parasites remained mononuclear; in contrast, following AP2XII-1/AP2XI-2_iKD depletion, a marked shift in nuclear architecture was observed. Enlarged single nuclei or alternatively multinucleated parasites increased in frequency, revealing the co-existence of two distinct “faiths” following DNA replication (either followed or not by immediate nuclear partitioning). Quantification over time revealed that zoites with multinucleated cells are more prevalent, at around 20%, than the ones with enlarged nuclei, with around 5%, at 12 and 24 h post-IAA. By 36 h, the percentage of enlarged nuclei stays relatively constant, but the number of cells exhibiting multiple nuclei increases with a concomitant decrease in mononucleated cells (Fig. 3C).

Two distinct scenarios likely contribute toward the increase in ploidy detected by flow cytometry (enlarged nuclei and multinucleated cells). By 48 h, however, when most parasites have transitioned to merozoites, the vast majority of cells once again contain a single “normal size” nucleus, coinciding with a return to the expected 1N ploidy. Overall, we observed two distinct but overlapping phenomena during differentiation: parasites exhibiting enlarged nuclei and parasites with multiple nuclei within a single cell.

The presence of enlarged and multiple nuclei per cell prompted us to ask whether an increment in nuclear size and likely DNA content is mechanistically linked to the expansion of key division machinery, including centrosomes and kinetochores. In addition, we wondered how a potentially polyploid nucleus temporally and mechanistically relates to the formation of a multinucleated syncytium. To tackle these questions, we resorted to TgCentrin1, a *bona fide* marker of the centrosome in *T. gondii* (35). Unlike the canonical cell cycle, in which the duration of the sequential stages allows distinction of phases by microscopy, the short duration of the *T. gondii* cell cycle and the lack of chromatin condensation preclude fine temporal resolution of immediate processes. S-phase entry is temporally coupled to the duplication of the TgCentrin1 signal, serving as an indirect proxy for assessing DNA replication (36).

**Fig 4:**
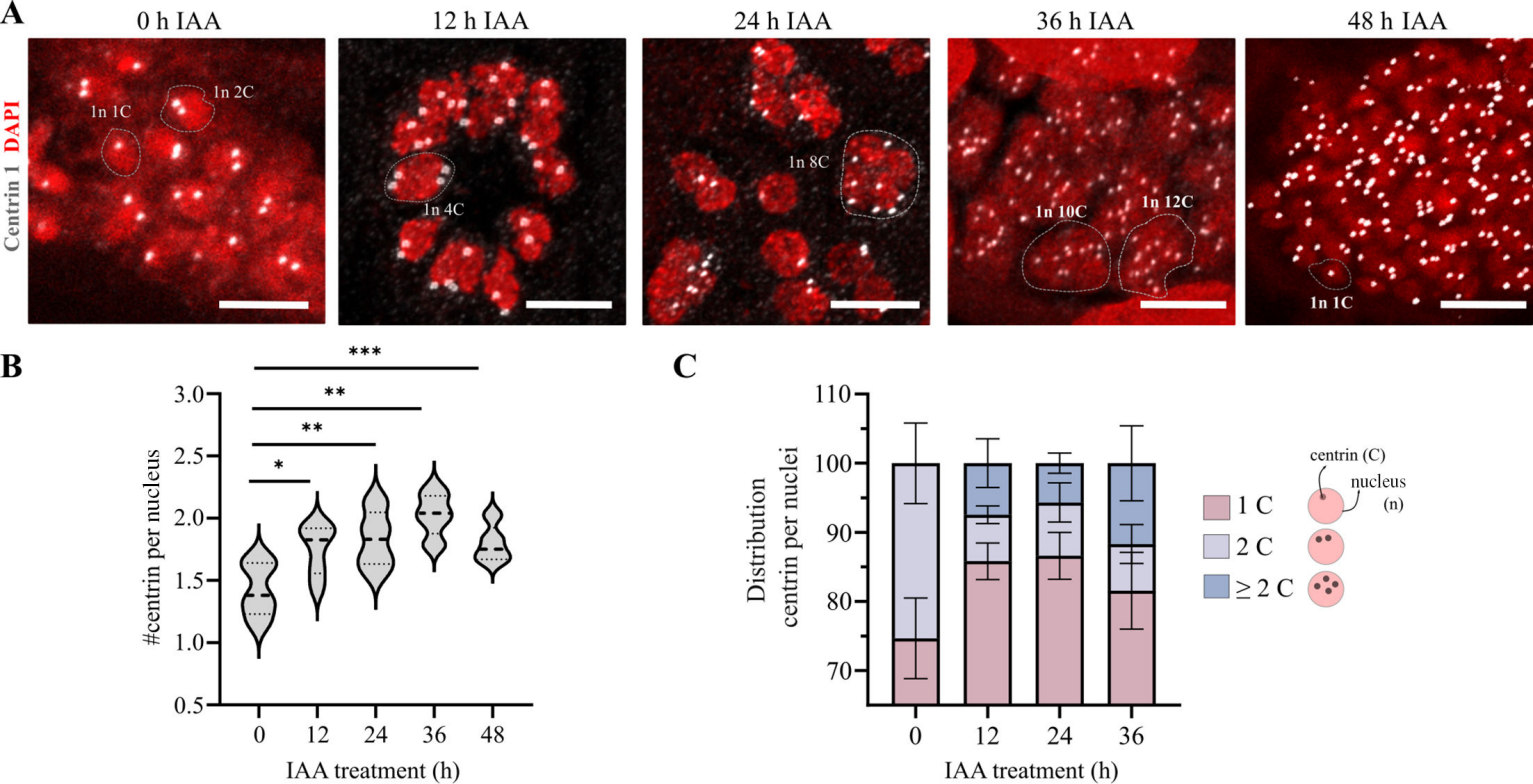
Observation of the centrosome/nucleus ratio upon AP2XII-1 and AP2XI-2 co-depletion. (**A**) Representative IFA from the different time points upon IAA addition, stained with anti-Centrin1 (gray), a *bona fide* marker of the centrosome, and DAPI (nuclear marker; red). Fluorescence confocal images shown are maximum intensity projections of z-stacks spanning the entire parasite. The scale bar corresponds to 5 μm. The letter n represents the number of nuclei, and C represents the number of centrin. (**B**) The total number of Centrin1 dots per nucleus (DAPI+) was quantified at different times following IAA addition to the growth media. Violin plots correspond to the mean of 500 to 1,000 nuclei per replicate, in five independent replicates, and represent the data distribution, with the median indicated by the central dotted line. Statistical evaluation was conducted using one-way ANOVA, followed by Tukey’s multiple comparison test, where *P < 0.05, **P < 0.01, and ***P < 0.001. (**C**) Distribution of nuclei (DAPI+, n) exhibiting 1, 2, or more than 2 associated centrin foci (C). The average is represented with the standard error of the mean. Note that the quantification for 48 h is not shown, as centrin foci could not be unequivocally assigned to individual nuclei.

To monitor nuclear division dynamics, we co-stained parasite DNA, its nucleus, and TgCentrin1. Cells divided by endodyogeny normally exhibit one detectable centrin per nucleus, corresponding to a cell in G1, while cells in early S-phase and through cell division exhibit two. On average, a population of asynchronous cells divided by endodyogeny exhibited 11 centrins/cell (37). Consistently, when cells are undifferentiated, we observed this expected ratio (Fig. 4A and B). A sustained increase in the average number of centrin signals per cell was observed up until 12 h post-IAA addition (Fig. 4B and C). Given that multiple nuclear states coexist at a given time point, we finely mapped the relative distributions of centrin per nucleus at each time. For all time points following IAA, we observed an increase in nuclei exhibiting four or more associated centrin signals. The percentage of nuclei exhibiting four or more associated centrin signals peaked at 36 h post-IAA addition (Fig. 4C). We also observed that contrary to synchronously dividing tachyzoites, parasites housed in the same parasitophorous vacuole (PV) showcase heterogeneous centrin/nucleus ratios, suggesting asynchronous cell divisions within individual PVs (Fig. 4A).

The presence of multiple centrin foci per nucleus supports a model in which nuclei undergo a transient polyploid stage prior to segregation. However, whether centrin duplication indeed correlates with the onset of DNA duplication in the pre-sexual stages studied herein has not been addressed. In addition, our experimental setup precludes us from simultaneously interrogating membrane components (i.e., daughter cell assembly). To address these limitations, we resorted to observing the kinetochores. In *T. gondii,* centromeres remain clustered in a single focus during G1 and duplicate into two distinct foci upon S-phase entry (38). Kinetochores follow the same kinetics (27), making the number of kinetochore foci a reliable proxy for DNA replication. Here, we used an anti-NDC80 antibody, which recognizes *Tg*NDC80, a component of the outer kinetochore plate in *T. gondii* (27), to monitor nuclear ploidy, in addition to anti-IMC1 to simultaneously observe daughter cell assembly.

**Fig 5:**
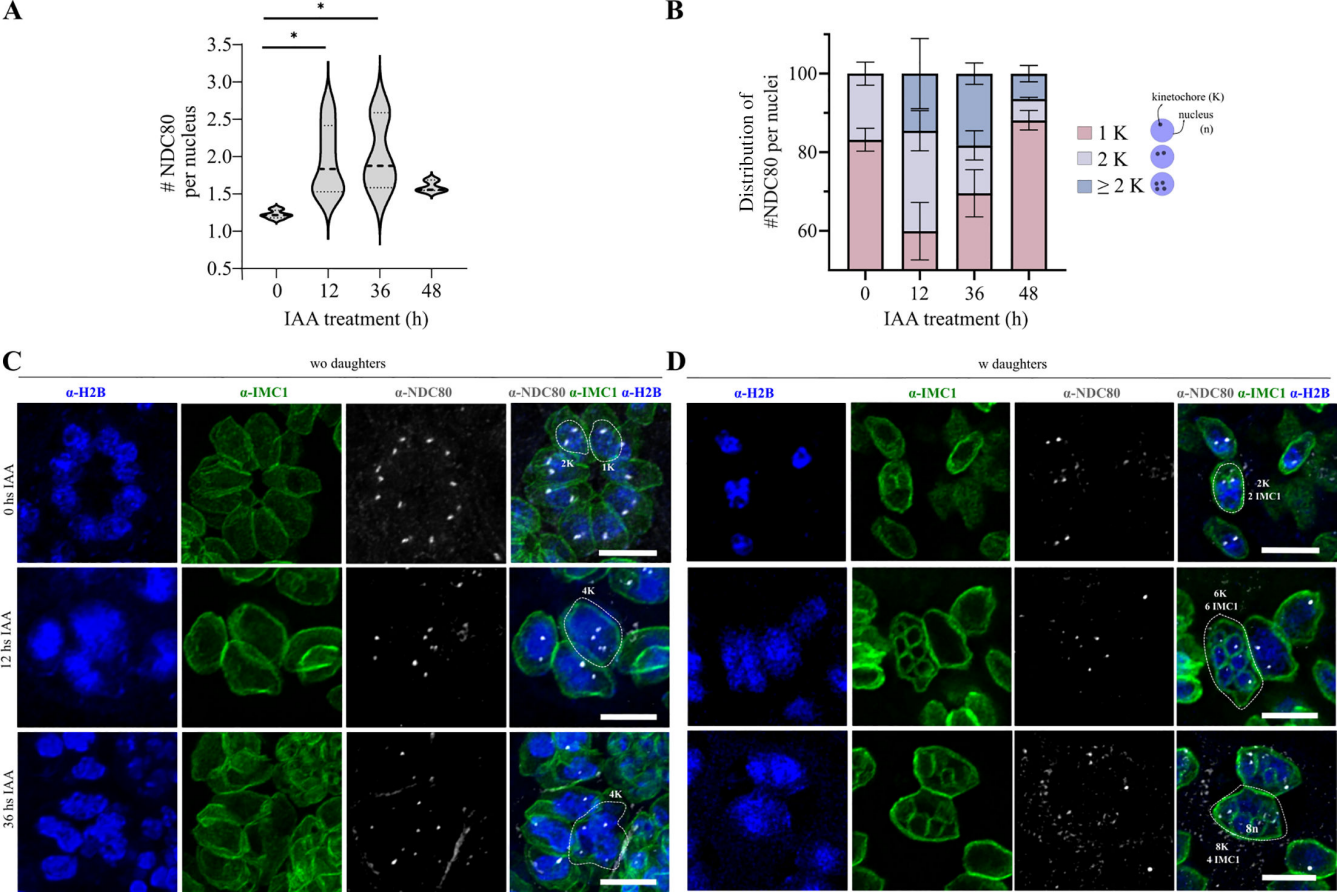
Observation of the kinetochore/nucleus ratio upon AP2XII-1 and AP2XI-2 co-depletion. (**A**) Quantification of the total amount of kinetochore (NDC80) per nucleus in the different times of merogony induction. Between 50 and 60 nuclei were quantified at least in three different replicates. Violin plots represent the data distribution, with the median indicated by the central dotted line. Statistical evaluation was conducted using one-way ANOVA, followed by Tukey’s multiple comparison test, where *P < 0.05. (**B**) Distribution of the number of kinetochore (K) as NDC80 dots per nucleus (H2Bz+, N) with 1, 2, or more than 2K per nucleus. The average is represented with the standard error of the mean. Note that the quantification for 24 h was not attained. (**C and D**) Representative IFA from the different time points used to induce merogony. Parasites were stained with anti-IMC1 (green), anti-NDC80 (gray), and anti-H2Bz (blue). A maximum intensity projected z-stack spanning the entire parasite is shown. The scale bar represents 5 μm. Nuclei without (**C**) or with (**D**) daughter assemblies are represented.

At 0 h, nuclei exhibit either one or two dots of NDC80, corresponding to either unduplicated centromeric/kinetochore regions and a single complement of chromosomes, or a duplicated chromosome complement, respectively. On average, nuclei at this time bear 1.2 NDC80 dots. By 12 h post-AP2XII-1/AP2XI-2_iKD depletion, the average number of NDC80 dots per nucleus increased to approximately 2, and this increase is sustained at 36 h (Fig. 5A). Detailed analysis of the number of NDC80 dots per nucleus at these time points indicated that approximately 15% of nuclei exhibited three or more NDC80 dots. By 48 h post-IAA addition to the growth media, over 90% of cells exhibited again 1 NDC80 per nucleus (Fig. 5B). To investigate when daughter assembly occurs with respect to transitions associated with nuclear partition and ploidy changes, we simultaneously followed daughter cells assembly using IMC1. We observed that polyploid nuclei exhibiting 4 NDC80 dots were generally associated with the presence of nascent daughter cells, suggesting that daughter cell budding initiates prior to karyokinesis (Fig. 5C). Interestingly, the number of daughter cells assembled did not match the number of NDC80 signals; instead, the number of NDC80 foci was often double that of assembling daughter cells. This suggests that a new round of DNA replication begins before daughter cell abscission, resulting in the inheritance of a 2n nucleus per daughter parasite (Fig. 5D).

As we observed that nuclei bearing duplicated DNA were inherited by daughter cells, we wondered how other organelles duplicated and partitioned during differentiation. Mitochondrial segregation is particularly conspicuous in *T. gondii*. The single mitochondrion of intracellular tachyzoites typically adopts a peripheral “lasso” shape surrounding the nucleus (39). During endodyogeny, mitochondrial segregation occurs at the very end of cell division by a last-minute switch in the membrane contact sites association from the mother to the newly emerging parasite pellicle (40, 41). In addition, close contacts between mitochondria and the nuclear membrane are observed in *T. gondii*, some of which are mediated by the nuclear pore components (42).

To explore mitochondrial segregation in the context of multiple daughter cells assembling simultaneously, we labeled the mitochondrial outer membrane protein *Mysterious* (Mys) using an anti-Mys antibody (39). Additionally, to visualize daughter cells, we co-stained parasites with an anti-acetylated tubulin antibody, which enables parasite microtubule-based structures’ detection (43). In cells with enlarged nuclei, the mitochondrion maintains the characteristic lasso-shaped morphology observed in non-dividing tachyzoites, encircling the nucleus. We observed that during early daughter cell formation, this morphology remains unchanged. However, at later stages of daughter cell assembly, and prior to daughter cell abscission, the mitochondria collapse toward the bottom of the mother cell to then be segregated into each emerging new parasite (Fig. S2).

**Fig 6:**
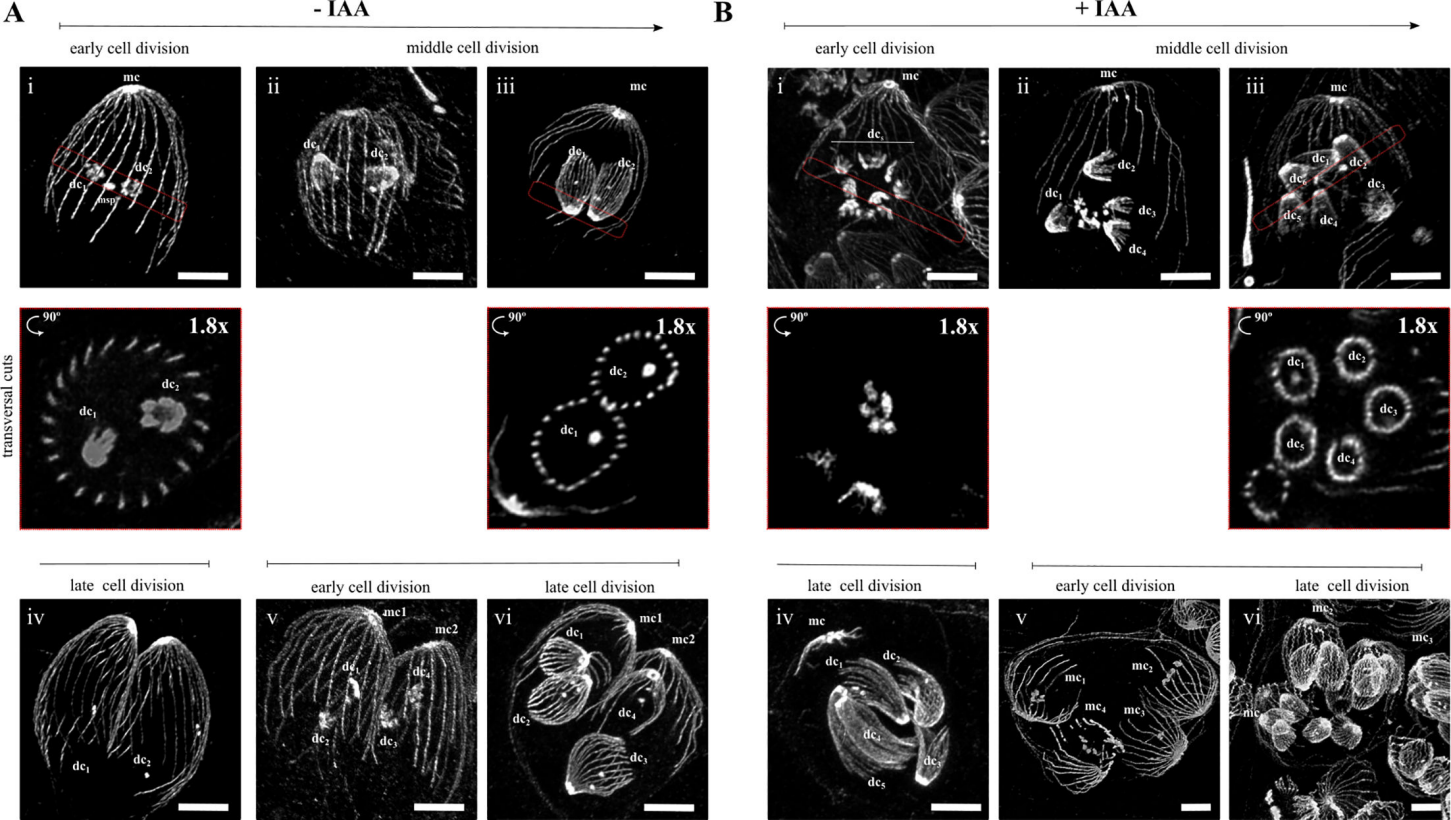
Dynamics of cell division of *T. gondii* by U-ExM. Mother and daughter cell cytoskeleton was observed and followed over time with anti-acetyl tubulin (gray) during the time-course of merogony by AP2XII-1 and AP2XI-2 co-depletion. (**A**) Endodyogeny of tachyzoites and (**B**) endopolygeny of meronts of *T. gondii*. Regarding the progression of the cell division, the parasites were grouped as early, middle, or late in advance in the cell division, and they are identified with roman numbers. Fluorescence confocal images represented are the maximum intensity projection of z-stacks. Red boxed regions indicate digitally magnified (1.8×) views highlighting the plane where subcortical microtubules initiate and develop during daughter cell formation. The scale bar represents 5 μm. mc, mother conoid or mother cell; dc, daughter cell; msp, mitotic spindle.

Recent studies have begun to elucidate the sequential steps involved in daughter cells scaffold assembly during endodyogeny. This process initiates with the formation of a “petal-like” arrangement of microtubule bundles, which eventually give rise to the complete daughter cytoskeleton (44, 45). Herein, we used ultrastructure expansion microscopy (U-ExM) in combination with an anti-acetyl tubulin antibody to analyze how daughter cells are formed in the context of differentiation. We achieved approximately 4-fold expansion, which allowed us to resolve the 22 sub-cortical microtubules expected in both non-dividing and daughter cells of dividing parasites at time 0 (Fig. 6A, i and iii). Upon IAA addition, we consistently observed asynchronous parasite division within individual PVs, as reflected by the different sizes of developing daughter cells (Fig. 6B, i-v, and Fig. S3). Considering that the centrosome is the platform upon which daughter cells are assembled, this observation is consistent with the variable number of centrin dots per nucleus detectable within a PV (Fig. 4). Interestingly, upon IAA addition, an enlarged nucleus and more than two nuclei, presumably corresponding to the polyploid stages, described in Fig. 3, were readily observable (Fig. S4 and S5). Cells exhibiting enlarged nuclei consistently showed the early budding of four daughter cells emerging from a central location adjacent to the mother’s nucleus (Fig. S5). During these early stages of daughter cell formation, we did not observe perturbation of the maternal cortical cytoskeleton. Daughter cell assembly seems to initiate by an akin process described for tachyzoites (44, 45), whereby five microtubule bundles precede the formation of the final 22 subpellicular microtubules in fully differentiated merozoites (Fig. 6B, i and iii). When the multiple daughter cells were almost emerged and assembled, karyokinesis was not apparent (Fig. S5), and the mother cytoskeleton was still visible (Fig. 6B, iv).

**Fig 7:**
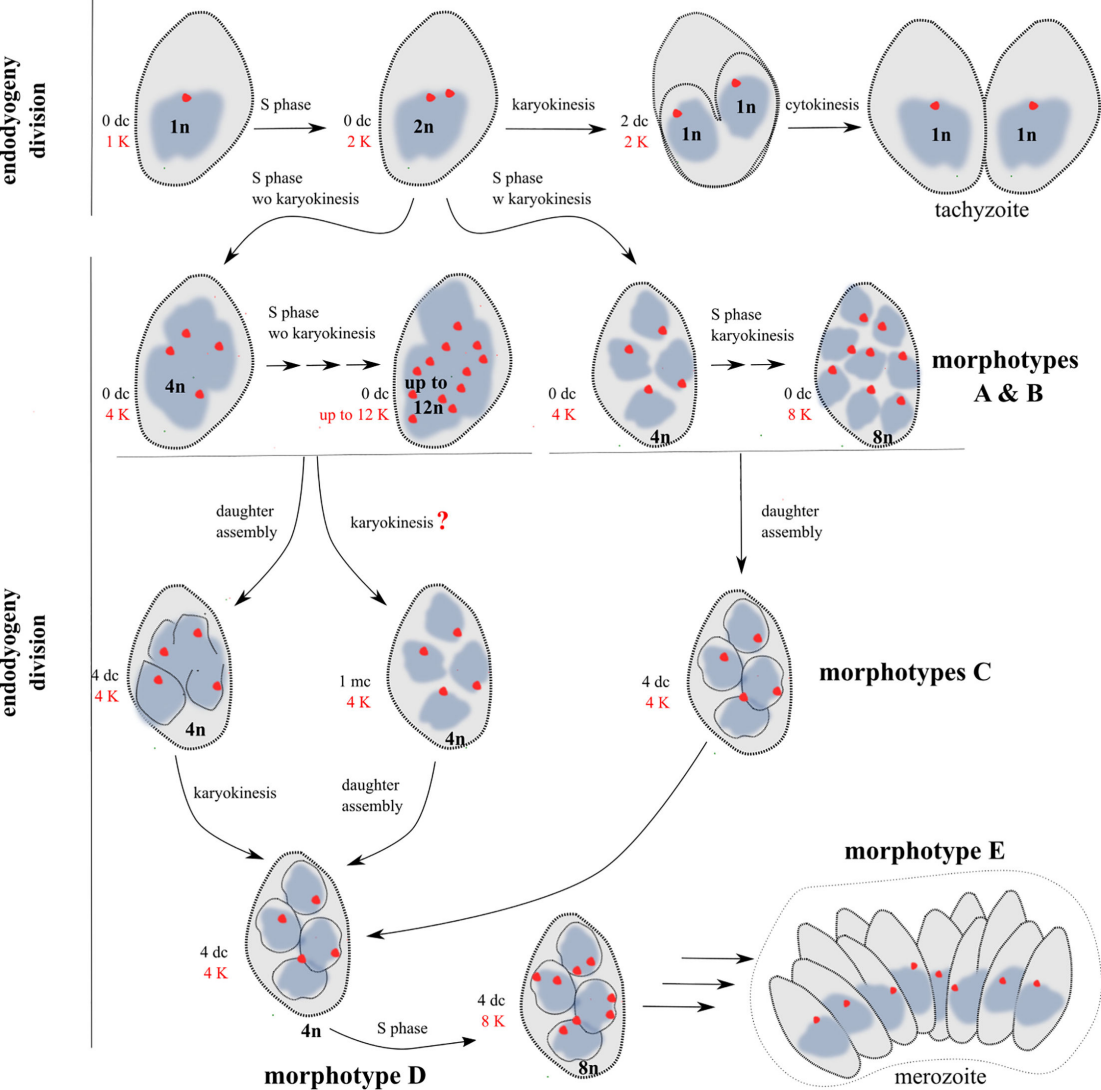
Representative model of the sequential cell division of *in vitro T. gondii* merozoites. In tachyzoites, replication occurs by endodyogeny, characterized by a single round of DNA replication, nuclear division, and the internal assembly of two daughter cells within the mother cytoplasm, with tight synchronization among parasites within the same parasitophorous vacuole. In contrast, differentiating merozoites undergo endopolygeny. Successive rounds of DNA replication can follow two alternative trajectories: either leading to progressive nuclear enlargement with increasing ploidy, or to nuclear fragmentation into multiple discrete nuclei prior to daughter cell budding (morphotypes A and B). Once a critical ploidy threshold is reached, multiple daughter cells assemble synchronously within the mother cell, either around a single polyploid nucleus or around multiple daughter nuclei (morphotype C). Centrosome amplification and kinetochore duplication increase progressively during differentiation and precede coordinated daughter cell assembly. Organelle segregation, including mitochondrial partitioning, occurs late in the budding process. Unlike tachyzoites, merozoite formation is characterized by heterogeneity in nuclear dynamics and asynchronous progression among parasites within the same vacuole. Morphotype D represents a late, pre-maturation stage in which daughter cells are fully assembled but retain a duplicated nuclear state, frequently exhibiting multiple NDC80 foci consistent with ongoing or recently initiated rounds of DNA replication prior to completion of cytokinesis. In contrast, morphotype E corresponds to fully formed mature merozoites, in which nuclear content has been reset following division, typically exhibiting a single NDC80 focus per nucleus that are individualized and densely packed within the parasitophorous vacuole. The division modes represented here are: DNA synthesis (S phase), karyokinesis, and daughter assembly. dcs, daughter cells; n, ploidy; K, number of kinetochores (a proxy of ploidy indicator).

Overall, our data suggest a model in which transition from tachyzoite to merozoite encompasses a switch from endodyogeny to endopolygeny, which may follow one of two alternative trajectories: parasites either progressively accumulate rounds of DNA replication in an unsegregated nucleus or undergo mitosis repeatedly prior to assembling daughters. Multiple discrete nuclei prior to daughter cell budding correspond with prior descriptions of morphotypes A and B. Once a critical ploidy threshold is reached, multiple daughter cells seem to assemble synchronously within the mother cell, either around a single polyploid nucleus or around multiple daughter nuclei. The latter is compatible with the morphotype C morphology description. Morphotype D represents a late, pre-maturation stage in which daughter cells are fully assembled but, our data suggest, retaining a duplicated nuclear complement, frequently exhibiting multiple NDC80 foci consistent with ongoing or recently initiated rounds of DNA replication prior to completion of cytokinesis. Finally, morphotype E likely corresponds to fully formed mature merozoites, in which nuclear content has been reestablished to a haploid content in individualized and densely packed within the parasitophorous vacuole merozoites. These data are summarized in Fig. 7.

## DISCUSSION

In this work, we aimed to characterize the poorly understood cell division modes used by *T. gondii* during its transition to merozoites, a key life stage preceding commitment to sexual stages. The interest driving this study lies in the mechanistic understanding of proliferation, which is critical to grasping pathogenicity and transmission. However, elucidating the proliferative cycles of human pathogens is logically capped by the accessibility to all different life stages. Non-accessible life stages hold potential for concealed drug targets due to our limited understanding of their biology. The discovery of the regulators triggering *in vitro* pre-sexual differentiation in *T. gondii* has tremendously advanced in the last few years, providing unprecedented access to these stages *in vitro*.

Herein, we leveraged the co-depletion of AP2XII-1 and AP2XI-2 to dissect nuclear and cytoskeletal events underlying differentiation from tachyzoites to merozoites. This approach allowed us to define distinct intermediate stages, which had been previously molecularly identified by expression of stage-specific markers and temporal enrichment during differentiation. Specifically, previously identified B-type morphotypes predominate at 12 h post-AP2XII-1/AP2XI-2_iKD co-depletion, while C and D morphotypes are more frequently observed at 24 h. By 48 h, fully differentiated merozoites became the dominant population (21). Our analyses reveal that time points in which early stages of differentiation are enriched showcase increased frequencies of parasites bearing enlarged nuclei, whereas multinucleated cells become more prevalent at later stages. Enlarged nuclei peak during the transition from B-type to C/D type morphotypes, whereas multinucleated cells are more frequently associated with the stages preceding mature merozoites. These shifts in nuclear organization were accompanied by distinct dynamics in daughter cell assembly. The highest frequency of parasites assembling daughter cells occurred during the multinucleated stages. Notably, the proportion of parasites forming multiple daughter cells also increased significantly when comparing early differentiation stages to undifferentiated tachyzoites, suggesting that daughter cell assembly at this stage is uncoupled from nuclear mitosis. While Antunes and colleagues (2024) reported daughter cell formation at the surface of the mother cell using electron microscopy, our observations were restricted to internal daughter cell budding, consistent with the endopolygenic mode of division.

Differences in nuclear dynamics and daughter cell formation reflect distinct coupling kinetics between DNA replication, nuclear mitosis, and budding. These features distinguish the three main division modes described in *T. gondii* and other coccidia: endodyogeny, schizogony, and endopolygeny (3). In schizogony, daughter cells bud from the periphery of a polyploid syncytial mother cell. In contrast, endopolygeny involves the synchronous assembly of multiple daughter cells within the cytoplasm of a mother cell, either around a single polyploid nucleus or following mitosis into multiple nuclei. Seminal electron microscopy studies *in vivo* reported schizogony as the division mode used by *T. gondii* to generate merozoites (46–49). However, Ferguson and collaborators (1974) also proposed endopolygeny as the mechanism of merozoite formation, describing geometric nuclear expansion (8–16 progeny per endopolygenic cycle) (17). Supporting this, similar patterns of internal daughter cell formation have been reported in pre-sexual forms of the closely related coccidians *Eimeria* and *Cystoisospora,* where merozoites are formed by daughter cells assembling adjacent to the nucleus rather than at the mother cell’s periphery (50, 51).

Herein, we observed the synchronous formation of multiple daughter cells originating both from a single polyploid nucleus or from multiple daughter nuclei, indicating that both nuclear configurations are compatible with endopolygenic budding. Daughter cell assembly proceeded in a highly coordinated fashion, with all daughters emerging simultaneously, even when associated with separate nuclei. This suggests the existence of a shared soluble regulator within the mother cell that orchestrates the timing of budding across all nuclei.

To probe the link between nuclear ploidy and cell division, we tracked DNA content by propidium iodide staining, followed by flow cytometry, observed centrosomes using Centrin1, and monitored kinetochore duplication via NDC80. Centrin foci progressively accumulated, reaching a peak at 36 h post-IAA addition, coinciding with the transition from C/D morphotypes to merozoites. This trend closely parallels the findings of Antunes and colleagues, who reported qualitatively centrin accumulation as early as 16 h post-AP2XII-1/AP2XI-2_iKD co-depletion. Kinetochores followed a similar trend, with daughter cell assembly typically starting only when nuclei reached at least more than 2N DNA content.

The gradual increase in DNA content and both centrin and kinetochore signals support a model of successive, rather than simultaneous, rounds of DNA replication. However, we noted substantial heterogeneity among parasites under identical conditions. Individual zoites showed variable centrin foci per nucleus and exhibited a broad range in daughter cell output—from more than 2 up to 12 daughters—whereby asynchronous cell division was apparent even within the same parasitophorous vacuole. This asynchrony likely stems from differences pre-established during the inherently asynchronous tachyzoite stage, whereby only a fraction of the population is undergoing cell division at any given time point. Similar developmental variability has been reported *in vivo* during naturally occurring pre-sexual stages (52).

To gain deeper insights into division dynamics, we used expansion microscopy following AP2XII-1/AP2XI-2_iKD co-depletion. We observed that while individual parasites formed daughters synchronously within a given mother cell, parasites within the same vacuole were often at distinct stages of division. This contrasts with tachyzoite replication, where parasites within a parasitophorous vacuole are typically tightly synchronized. While slight variations in the exact timing of S-phase entry for parasites within the same vacuole have been recorded in 7%–30% of cases (36), the timing of daughter cell assembly is generally coordinated. Previous work proposed that general synchronization in tachyzoite daughter cell formation is maintained through cytoplasmic connections provided by actin-based basal end structures (53). *In vivo* formed merozoites have been described by electron microscopy to remain attached to a small amount of residual cytoplasm at the posterior end (54). However, our observations suggest that the more complex events leading to endopolygeny introduce variability in division timing among individual parasites within the same PV that consequently cause a perceivable desynchronization of daughter cell assembly dynamics.

Coordinated segregation of organelles is essential for producing viable daughter cells, especially when multiple progenies emerge simultaneously. Apicoplast segregation during tachyzoite replication is well documented, and both *in vitro* and *in vivo* studies of merozoites have shown that the apicoplast elongates and branches in coordination with nuclear replication (21, 55). Mitochondrial segregation during endopolygeny is less well understood. Ferguson et al. (17) noted an increase in mitochondria size and number in *in vivo*–generated merozoites, often located at the cell periphery. Here, we found that the mitochondrion maintained a lasso-like morphology throughout most of the differentiation process. Only shortly before abscission did the mitochondrion collapse and undergo partitioning into individual daughters. This is consistent with observations in dividing tachyzoites (39, 56) and suggests a conserved mechanism of mitochondrial segregation across division modes.

Daughter cell formation in *T. gondii* is driven by the sequential assembly of cytoskeletal elements, including the apical complex and cortical microtubules. In endodyogeny, the apical polar ring nucleates the 22 microtubules that form the parasite’s cortical corset (57). This array is initially visible as a flower-like microtubule arrangement, as reported in recent studies (44, 45). Here, we observed a similar early-stage structure, with 5-6 microtubule clusters forming around a presumptive conoid, eventually organizing into the typical 22-microtubule configuration. Contrary to what is observed for endodyogeny of tachyzoites, where cytoskeleton disassembly of the mother overlaps with daughter emergence during endopolygeny (3, 58), we observed that the mother’s cytoskeleton was preserved until daughter emergence. This is consistent with the observations reported by Antunes et al. (21), who found intact maternal conoids during late daughter assembly stages.

A limitation of our study is that the parasites analyzed undergo a direct transition from tachyzoites to merozoites, a route that may not fully recapitulate the canonical developmental trajectory, which postulates that merozoites are first generated from bradyzoites. However, recent single-cell transcriptomic analyses have challenged this established paradigm, suggesting that bradyzoites may not convert directly into fully committed merozoites, but instead transit through an intermediate transcriptional state, which potentially resembles tachyzoite-like or mixed-stage programs prior to activation of the merozoite gene expression network (59). In this context, our system may capture features of these transitional states, providing insight into early events in merozoite development. Additionally, our work uncovers the previously unappreciated coexistence of polyploid forms that undergo budding from a single enlarged nucleus and those that bud from individualized nuclei. We reckon that these alternative trajectories may reflect the emergence of distinct subpopulations early along the differentiation track. We speculate that such divergent programs, driven by yet undefined regulatory mechanisms, could underlie subsequent differences in cell fate decisions, including the commitment of merozoites to macro- versus micro-gamete lineages. Exploring these possibilities will be an important direction for future work.

## SUPPLEMENTAL MATERIAL

Supplemental material mbio.02440-25-s0001.docx Supplemental figures and tables.

ASM does not own the copyrights to Supplemental Material that may be linked to, or accessed through, an article. The authors have granted ASM a non-exclusive, world-wide license to publish the Supplemental Material files. Please contact the corresponding author directly for reuse.
