# Supplementary material for: Proliferating toward sex: characterization of cell division of Toxoplasma gondii’s pre-sexual stages
Source: mBio. 2026 May 19;17(6):e02440-25. doi: 10.1128/mbio.02440-25 (PMC13251383; doi:10.1128/mbio.02440-25)
Supplement: Supplemental material — Supplemental figures and tables. [file mbio.02440-25-s0001.docx]

**Supplementary data**

**
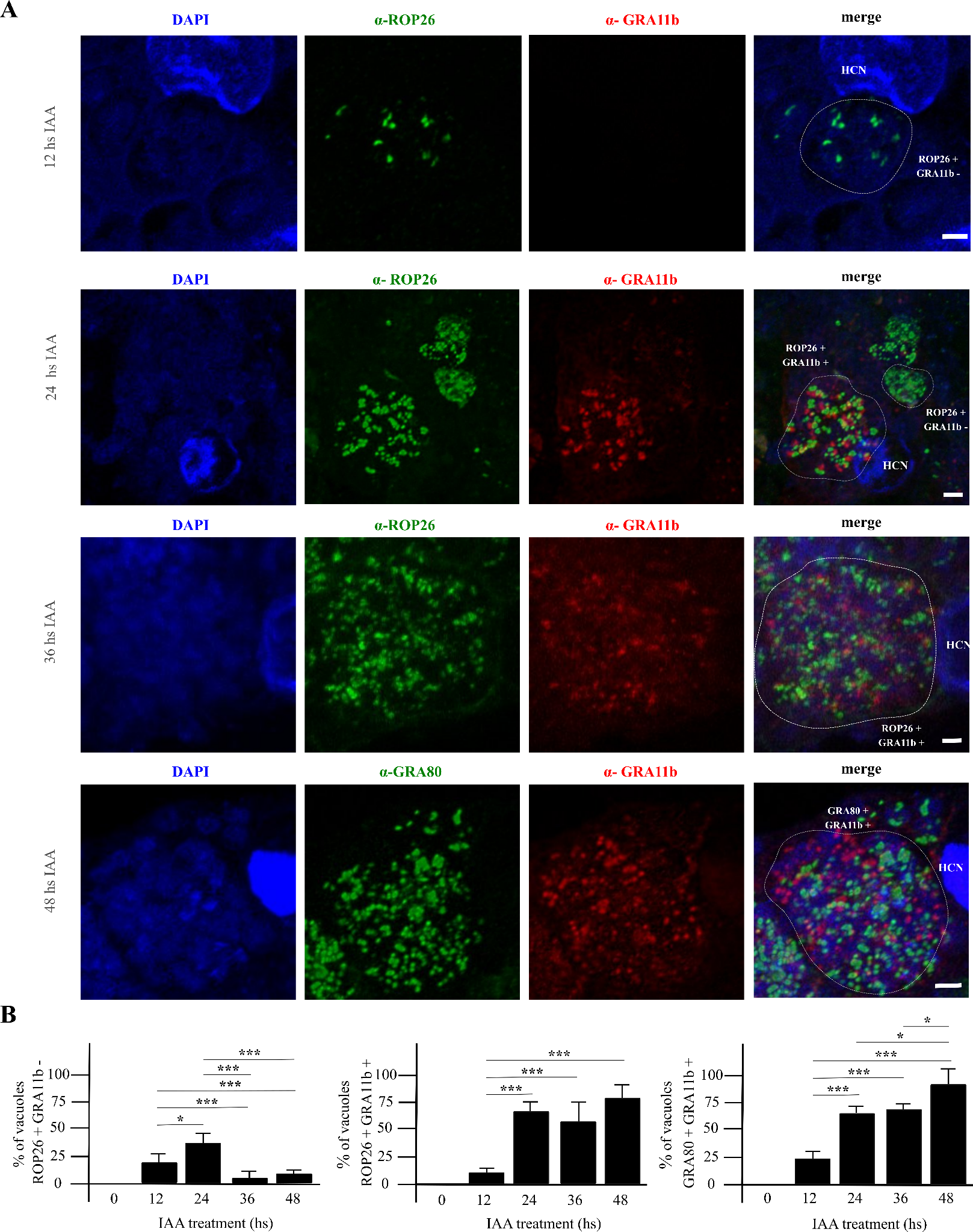
**

**Supplementary Figure 1. Time-course analysis of merozoite marker expression following AP2XII-1 and AP2XI-2 simultaneous depletion.** **A**. Representative images of immunofluorescence assays of the different time points following IAA addition. Parasites were stained with α-ROP26 (green), or α-GRA80 (green), α-GRA11b (red), and DAPI (blue), as indicated. Fluorescence confocal images shown correspond to maximum intensity projection of z-stacks encompassing the entire vacuole. HCN: host cell nucleus; PV: parasitophorous vacuole. Scale bar = 2 μm. **B.** Quantification of the relative abundance of ROP26, GRA80, and GRA11b expression profiles over time. Data represent mean ± s.d. of vacuole staining for ROP26+/GRA11b- (morphotype B), ROP26+/GRA11b+ (morphotypes C and D), or GRA80+/GRA11b+ (merozoite) over the total of vacuoles formation from five experiments (n ≈ 30 vacuoles/replicate). Statistical evaluation was conducted using one-way ANOVA, followed by Tukey’s multiple comparison test, where * p < 0.05, ** p < 0.01, and *** p < 0.001**.**

**
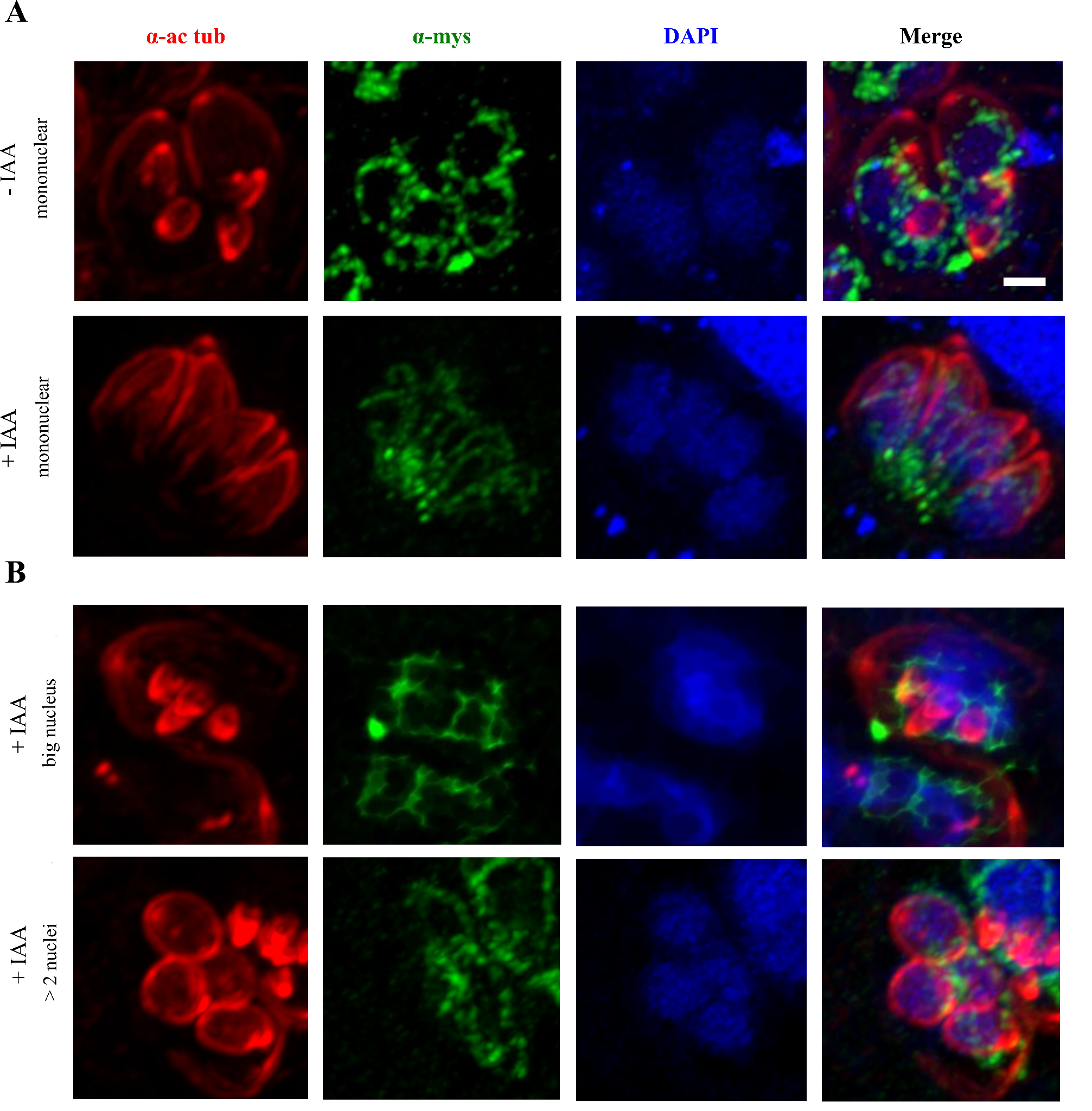
**

**Supplementary Figure 2. Mitochondria dynamics changes along differentiation. A.** IFA from mononuclear parasites with or without IAA addition (**A**) and either enlarged nucleus or multiple nuclei after 12 to 48 hs of AP2XII-1 and AP2XI-2 co-depletion (**B**). α-ac tub (red), α-mys (green), and DAPI (blue). The scale bar represents 2 μm.

**
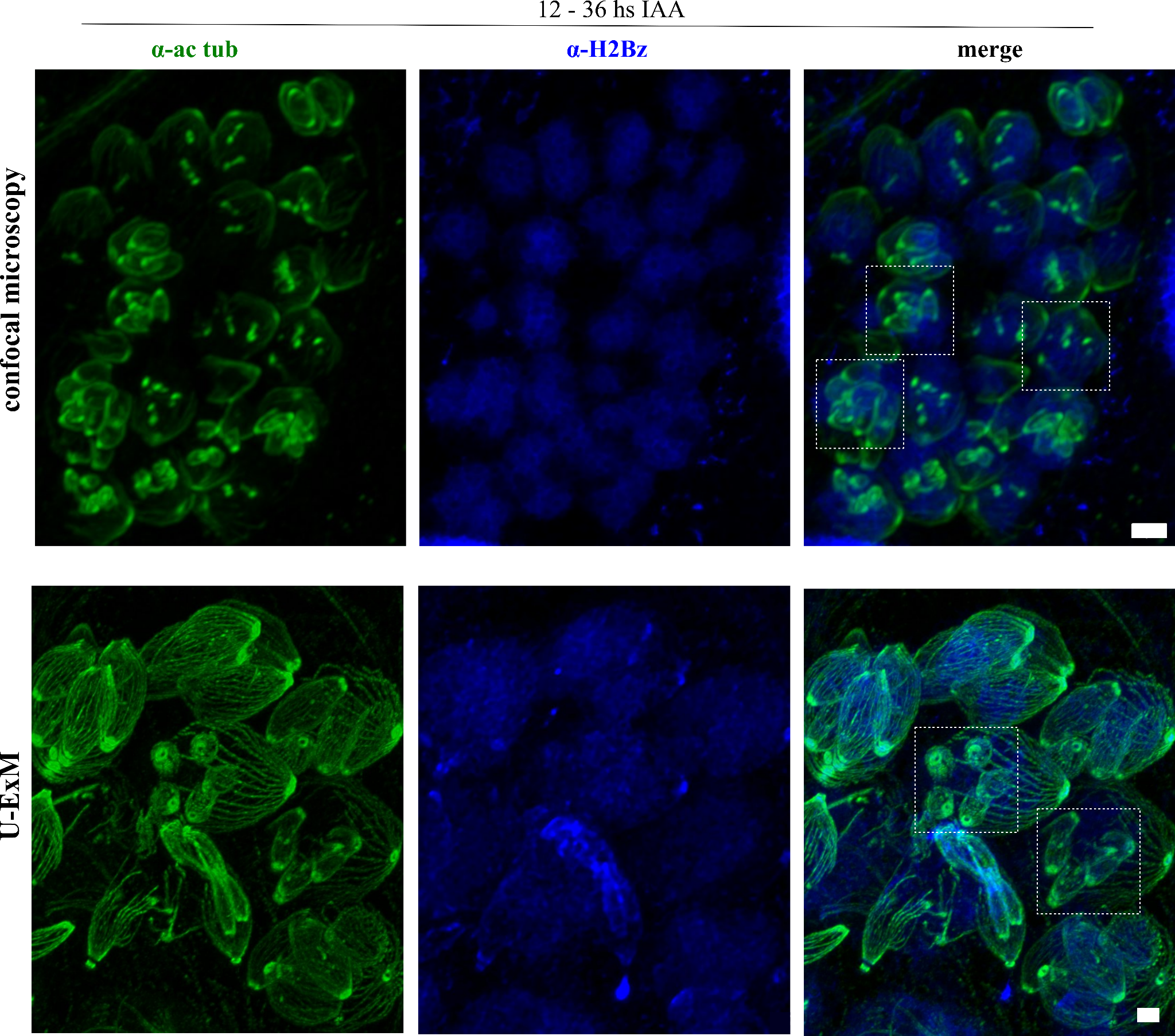
**

**Supplementary Figure 3. Imaging of AP2XII-1 and AP2XI-2 co-depletion using conventional microscopy and U-ExM.** Parasites were imaged 36 hs after IAA treatment. The antibodies were α-ac tub (green) and α-H2Bz (blue). The white boxes represent different grades of daughter cells emerging from a unique nucleus. Fluorescence confocal images represented are the maximum intensity projection of z-stacks. Scale bar = 5 μm.

**
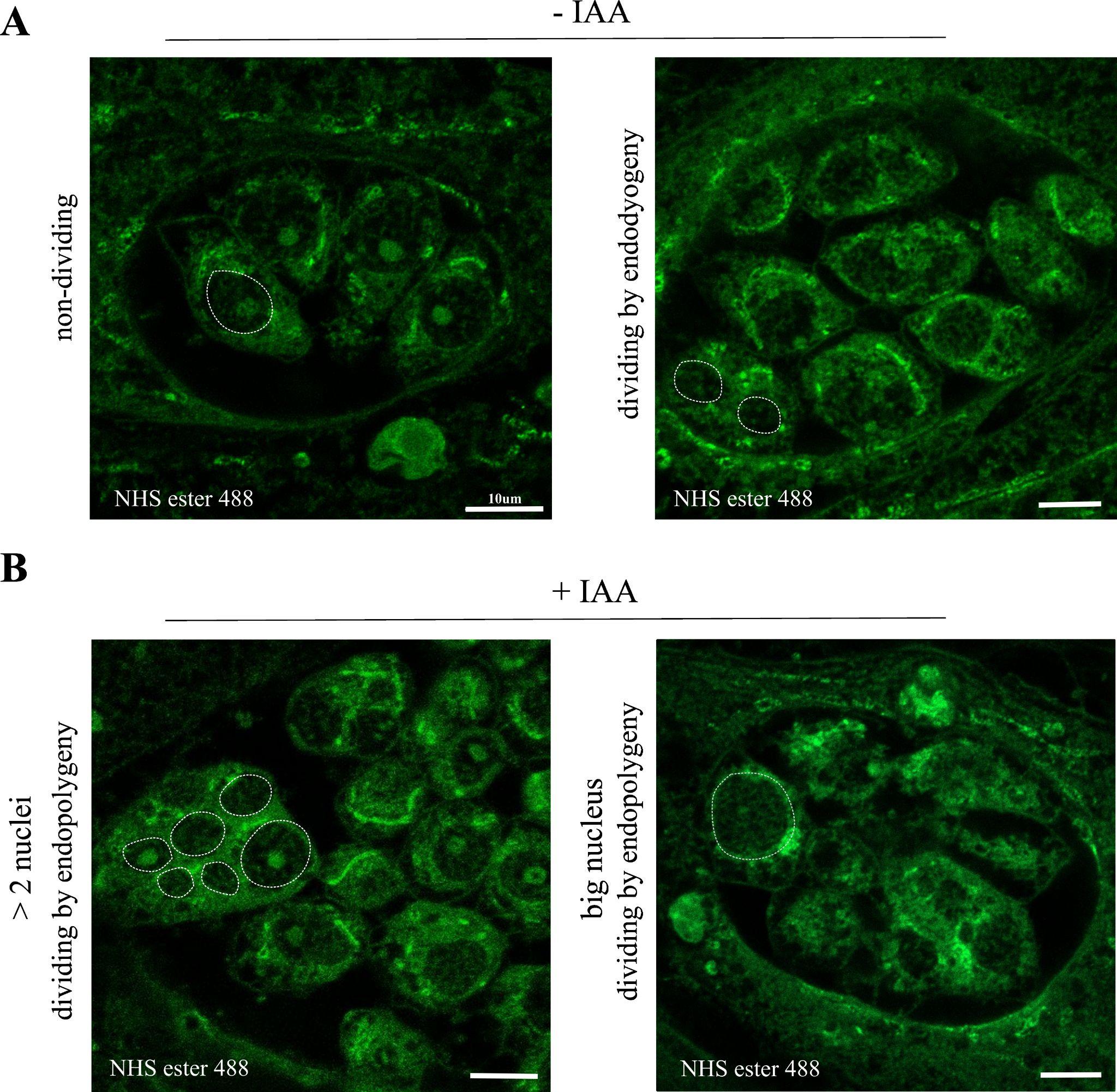
**

**Supplementary Figure 4. Imaging of AP2XII-1 and AP2XI-2 co-depletion using U-ExM.** Parasites were imaged without (**A**) and with IAA addition (**B**) and stained with NHS ester 488 nm. White circles highlight the different nucleus sizes. Scale bar = 10 μm.

**
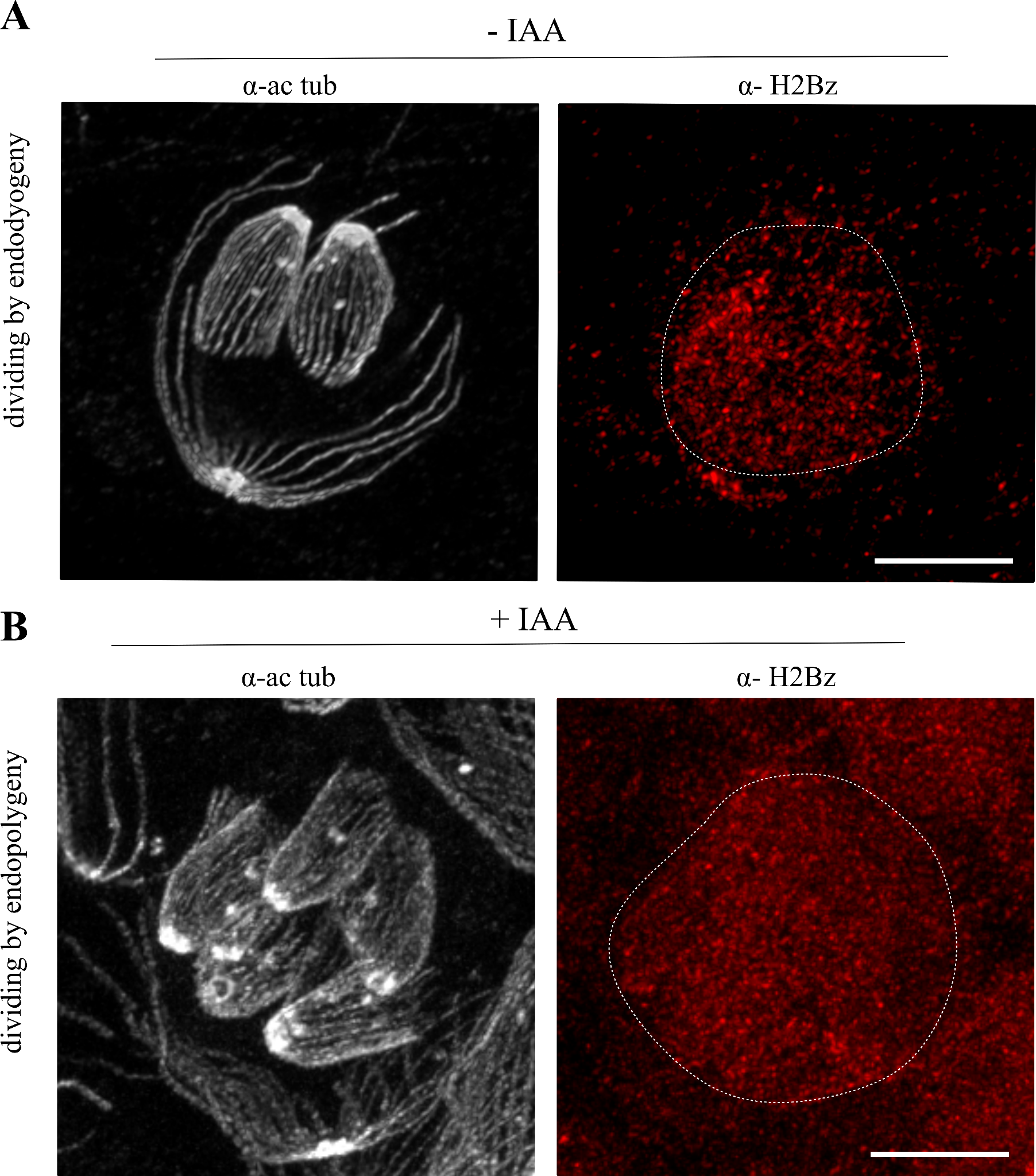
**

**Supplementary Figure 5. Imaging of AP2XII-1 and AP2XI-2 co-depletion using U-ExM.** Parasites were imaged without (**A**) and with IAA (**B**) and stained with α-ac tub (grey) and α-H2Bz (red). White circles highlight the nucleus of mothers assembling daughters either during endodyogeny or endopolygeny. Scale bar = 5 μm.
